# Boosting NAD+ levels through fasting to aid in COVID-19 recovery

**DOI:** 10.3389/fimmu.2024.1319106

**Published:** 2024-02-14

**Authors:** Rachmad Anres Dongoran, Meity Mardiana, Chih-Yang Huang, Jiro Hasegawa Situmorang

**Affiliations:** ^1^ Directorate of Drug Registration, Indonesian Food and Drug Authority, Jakarta, Indonesia; ^2^ Center for Chinese Studies, National Central Library, Taipei, Taiwan; ^3^ Institute of Medical Sciences, Tzu Chi University, Hualien, Taiwan; ^4^ Cardiovascular and Mitochondrial Related Disease Research Center, Buddhist Tzu Chi General Hospital, Buddhist Tzu Chi Medical Foundation, Hualien, Taiwan; ^5^ Graduate Institute of Biomedical Sciences, China Medical University, Taichung, Taiwan; ^6^ Department of Medical Research, China Medical University Hospital, China Medical University, Taichung, Taiwan; ^7^ Department of Biotechnology, Asia University, Taichung, Taiwan; ^8^ Center of General Education, Buddhist Tzu Chi Medical Foundation, Tzu Chi University of Science and Technology, Hualien, Taiwan; ^9^ Center for Biomedical Research, National Research and Innovation Agency (BRIN), Cibinong, Indonesia

**Keywords:** calorie restriction, COVID-19, fasting, influenza virus, long covid, SARS-CoV-2

## Introduction

Pharmacological therapy has played a vital role in combating various diseases, with dozens of novel drugs being approved each year. In the wake of severe acute respiratory syndrome coronavirus 2 (SARS-CoV-2) or COVID-19 outbreaks, scientists and researchers have been in a race against time to develop vaccines, discover new drugs, and repurpose old drugs for COVID-19. As of now, several drugs have been approved for the treatment of COVID-19 (1). COVID-19 is anticipated to transition into an endemic state, signifying its enduring presence within the population. However, this does not negate the potential for the virus to undergo evolutionary changes that could lead to sporadic outbreaks or seasonal surges. Even the World Health Organization (WHO) has recently issued standing recommendations that outline critical actions supporting the transition from emergency response to COVID-19 into strengthened and integrated infectious disease prevention and control programs (2). The goal is to prepare for potential worsening situations caused by new variants of the virus and to reduce the disease burden from COVID-19, which include addressing post-COVID-19 conditions such as long COVID.

Apart from pharmacological therapy which is primarily based on medication, a non-pharmacological intervention such as sleep improvement, dietary habits, exercise, and fasting has been has been introduced in managing various diseases and accelerating recovery (3). Fasting, with its historical roots dating back to the 5th century, is a practice that carries significance not only from religious perspectives but also due to its potential for enhancing overall well-being. Hippocrates, father of medicine of ancient Greece, advocated fasting as a therapeutic intervention for specific diseases. Extensive research conducted in the 20th century involving animals and humans has further illuminated the profound benefits associated with fasting, encompassing cancer prevention (4), weight loss (5), improved insulin sensitivity (6), and even increased lifespan (7). Notably, advancements in molecular analysis have provided insights into the underlying mechanisms through which these benefits are realized. Fasting has been found to effectively suppress genes implicated in diseases such as cancer, cardiovascular diseases, and metabolic disorders (7). This genetic modulation plays a crucial role in mitigating the risk and progression of these illnesses. Furthermore, fasting stimulates molecular metabolic pathways associated with anti-aging processes, particularly involving autophagy-related proteins that facilitate cellular rejuvenation and maintenance (8). Ongoing research is being conducted on drug development aimed at replicating the effects of fasting and promoting life extension. Compounds such as metformin, rapamycin, and nicotinamide are among the most extensively studied drugs in this context, demonstrating potential in enhancing longevity (9).

Despite the well-documented benefits of fasting, its potential as a therapeutic approach for aiding in illness recovery often remains unrecognized, leading to a dearth of research in clinical settings. Studies have demonstrated the effectiveness of fasting in preventing SARS-CoV-2 infection (10, 11). We propose that the accelerated recovery from COVID-19 through fasting is likely mediated by the increase in nicotinamide adenine dinucleotide (NAD+). We outline several underlying mechanisms to support this viewpoint. Our aim is to stimulate further research on the utilization of fasting or fasting-mimicking drugs as methods to enhance NAD+ levels, thereby potentially countering viral infections, particularly SARS-CoV-2.

## The essential role of NAD+ in immune cells combating COVID-19

NAD+ is a coenzyme and cofactor pivotal for cellular energy production, DNA repair, regulation of inflammation and oxidative stress. Additionally, NAD+ serves as a crucial substrate in various cellular signaling pathways, including those mediated by sirtuins, which are best known for their anti-aging effects. Viral replication is a complex process that demands significant energy and resources from host cells. Viruses often exploit cellular machinery to replicate their genetic material and produce new virus particles. NAD+ plays a pivotal role in supporting these processes. It serves as a coenzyme in numerous enzymatic reactions, including those essential for DNA and RNA synthesis of the virus. Research suggests that viruses, including coronaviruses like SARS-CoV-2, exploit NAD+ resources during their replication cycle. The virus can activate specific enzymes that utilize NAD+ to facilitate various steps of their replication (12, 13). This heightened demand for NAD+ could potentially diminish cellular NAD+ levels, leading to consequences beyond viral replication itself. Reduced NAD+ levels might compromise cellular energy production and impair the functioning of immune cells that are crucial for recognizing and combating viral infections (14). Given that both cellular function and viruses rely on NAD+ for their survival, the idea of increasing NAD+ could have both positive and negative implications. However, previous study have shown that replenishing NAD+ accelerates recovery from COVID-19 in mouse models (15)..

During COVID-19 infection, the respiratory system is primarily affected, leading to symptoms like persistent cough, shortness of breath, and sore throat. Interestingly, a significant number of COVID-19 patients display immunosuppression or a hypoinflammatory state, wherein immune cells like macrophages exhibit diminished responsiveness to the infection, marked by lower pro-inflammatory cytokines (16, 17). The function of immune cells, such as macrophages, is akin to a double-edged sword. When their levels are too low, the ability to combat infection worsen. Similarly, when their levels are too high, there is potential harm to healthy cells due to the increase in pro-inflammatory cytokines. Important to note that in the COVID 19, the macrophages also contribute to hyperinflammation in the lungs, further exacerbating the severity of the disease (18). The significance of NAD+ is crucial in this context, as a decrease in NAD+ levels can alter the polarization of macrophages towards a proinflammatory state (19). Additionally, insufficient NAD+ levels within macrophages can disrupt their metabolic processes, resulting in functional impairment and ultimately leading to macrophage death (20). This outcome results in an inadequate immune response against infections. Furthermore, the accumulation of deceased macrophages in the lung mucosa contributes to the buildup of extracellular matrix, potentially leading to irreversible lung fibrosis over time (21). While speculative, these mechanisms may offer insights into long COVID persistent symptoms like shortness of breath and shallow breathing. Approaches aimed at increasing NAD+ levels have been shown to restore proper functioning to macrophages within the immune system, regulating inflammation and the repair process (15, 22).

Lymphocytes, another essential component of the immune system, play a pivotal role not only in COVID-19 but also in overall viral infections. There are two types of lymphocytes: B cells and T cells. In the adaptive immune response, lymphocytes play a crucial role in recognizing and combating viruses, effectively destroying them, and possessing the ability to retain a memory of encountered viruses, ensuring a swift response upon future infections. During a viral infection, innate immune cells such as macrophages activate CD4+ T helper cells through antigen presentation. These activated helper T cells then release cytokines that activate cytotoxic CD8+ T cells. The activated helper T cells also interact with B cells, causing the differentiation of B cells into plasma cells capable of producing antibodies to combat the virus. Meanwhile, CD8+ T cells can directly recognize virus antigens expressed on MHC I molecules on the surface of virus-infected cells, leading to the induction of apoptosis in these infected cells. The activated B cells and T cells can also differentiate into memory B cells or memory T cells to ensure rapid neutralization of future infections (23). In the case of COVID-19, lymphocyte levels are decreased (24). Intriguingly, the count of lymphocytes serves as a robust predictor of disease severity and mortality in COVID-19 (25). Similar to macrophages, lymphocytes also rely on NAD+ for survival and normal function. Studies indicate that a decline in NAD+ levels in lymphocytes can disrupt their response to mitogens, leading to reduced proliferation and indicating impaired functionality (26). While boosting NAD+ is likely to enhance lymphocyte counts and function, no studies have been conducted to confirm this.

## Susceptibility to COVID-19 in elderly individuals and those with diabetes might be associated with low levels of NAD+

NAD+ levels decline with age, contributing to cellular dysfunction and susceptibility to various diseases (27, 28). Notably, aging is recognized as a significant risk factor for severe COVID-19 manifestations (29). Diabetes, likewise, presents a distinct challenge during COVID-19. Individuals with diabetes, whether it is type 1 or type 2, exhibit compromised immune responses and heightened inflammation, rendering them more susceptible to severe outcomes (30). NAD+ plays a multifaceted role in glucose metabolism, insulin sensitivity, and inflammation regulation. In type 1 diabetes, the low level of NAD+ primarily results from the activation of poly ADP‐ribose polymerase. Meanwhile, in type 2 diabetes, a larger contributing factor is the inhibition of adenine nucleotide monophosphate‐activated protein kinase (AMPK) activation (31). Thus, diminished NAD+ availability could potentially contribute to metabolic imbalances, further exacerbating the susceptibility of diabetic patients to severe COVID-19 complications. Further complexity arises from the interaction between SARS-CoV-2, the virus causing COVID-19, and NAD+ metabolism. The virus’s replication process demands NAD+ resources, potentially leading to a depletion of this vital coenzyme in infected cells (12, 13). This depletion might trigger a cascade of events, including compromised cellular functions and heightened inflammation, potentially culminating in severe lung injury and respiratory distress – hallmarks of severe COVID-19 cases. Hence, for these reasons, elderly and diabetic individuals are more prone to COVID-19.

## Fasting as a way to boost NAD+ levels and its potential to fight COVID-19

**Figure 1:**
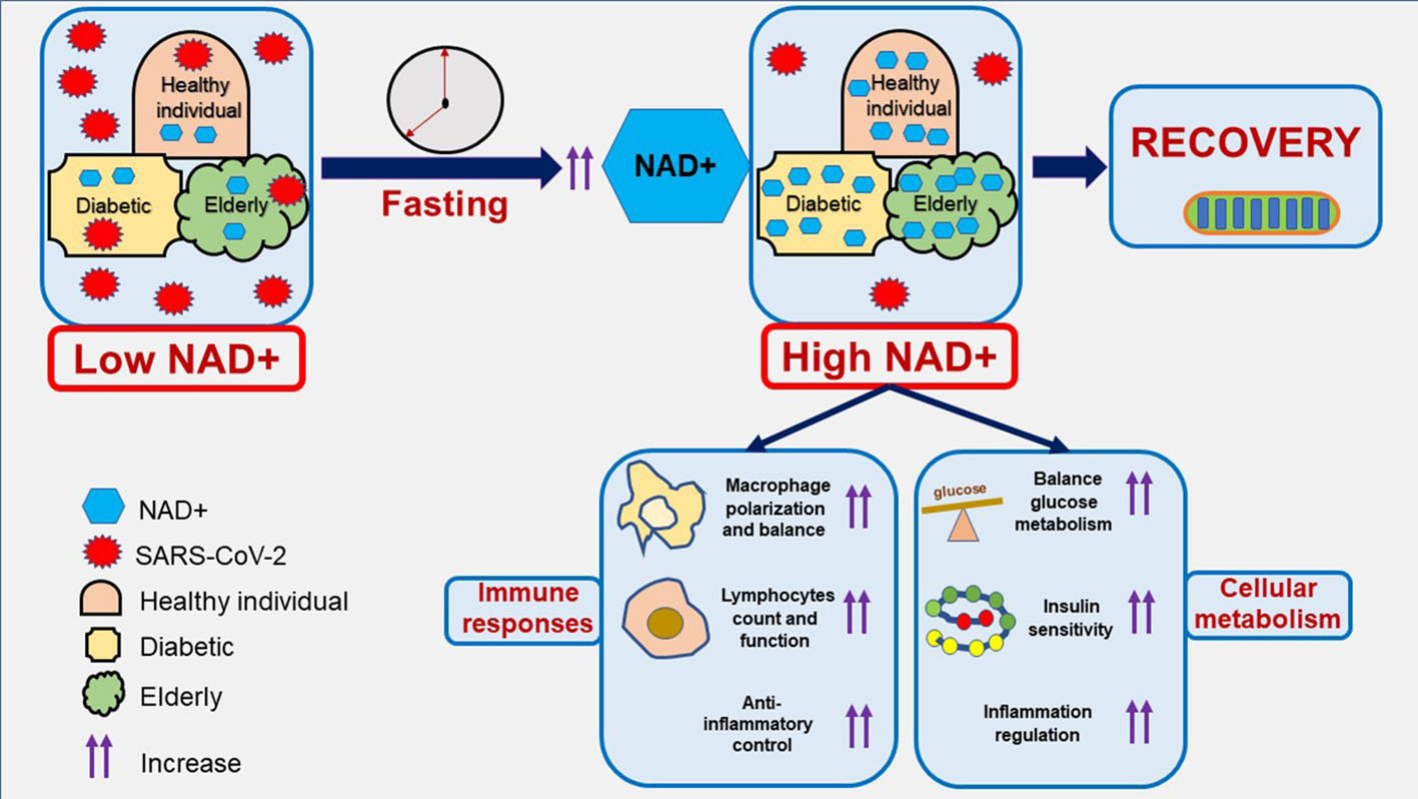
An illustration of boosting NAD+ levels through fasting to aid in COVID-19 recovery. NAD+ plays an essential role in immune cells combating COVID-19. The NAD+ level decreases in patients infected with COVID-19, whether they are healthy individuals, diabetics, or elderly people. Fasting is considered one of the strategies to increase NAD+ levels in combating COVID-19, thereby enhancing the immune system and metabolism. This approach represents a potential adjuvant therapy against COVID-19 and could aid in managing long COVID.

One intriguing aspect of fasting is its influence on cellular metabolism and the modulation of key molecules, including NAD+ (32). Moreover, fasting’s potential to mitigate the progression of viral infections, including COVID-19 caused by the SARS-CoV-2 virus, has been demonstrated in several studies (10, 11, 33). During fasting, cells undergo a metabolic switch from glucose metabolism to fatty acid oxidation and ketone utilization. This metabolic adaptation triggers pathways, such as sirtuins, that rely on NAD+ as a substrate. These result in postive feedback, wherein fasting can increase cellular NAD+ levels, potentially conferring cellular resilience to stress. The elevation of NAD+ induced by fasting might enhance antiviral defenses by bolstering immune cell function, which fights viral infection, and regulating inflammation (**Figure 1**). Consequently, this could alleviate the severity of COVID-19. However, translating the potential benefits of fasting into clinical applications warrants careful consideration. Fasting regimens vary widely, and their effects on different populations, such as the elderly and young person, including those with existing health conditions, are diverse. However, the effects of fasting in COVID-19 patients without preexisting health conditions have shown promising results (11). The potential of fasting to combat COVID-19 aligns with the broader concept of therapeutic interventions that target host cell mechanisms to control viral replication. While antiviral drugs directly target the virus, interventions that enhance host cell defenses could offer a complementary strategy. Fasting-induced elevation of NAD+ might represents a unique approach in this regard.

In addition, fasting-induced elevation of NAD+ could be a potential approach to address long COVID conditions. Long COVID or post COVID condition refers to a prolonged condition characterized by symptoms that persist or develop after the initial COVID-19 infection, affecting various organs with different underlying issues. Long COVID tends to occur more frequently in cases of severe COVID-19 infection, and to date, more than 200 different symptoms have been identified. Furthermore, the number of long COVID cases is on the rise, estimated to impact approximately 65 million individuals worldwide (34). Long COVID can be extremely debilitating; with each new infection or reinfection of COVID-19, there is a heightened risk of developing this condition, which in turn increases the likelihood of severe medical complications. It is important to note that current diagnostic and treatment options are often inadequate in managing long COVID. Nevertheless, researchers have made significant strides in understanding the various pathophysiological changes associated with long COVID, including a compromised immune system. In light of these findings, fasting-induced elevation of NAD+ presents itself as a potentially crucial strategy to bolster the immune system’s capacity to combat long COVID (35).

It is important to acknowledge that fasting is not without challenges. Prolonged fasting can lead to nutritional deficiencies and may not be suitable for everyone. In addition, it is imperative to acknowledge that fasting might not be tolerable for all individuals due to the sensation of hunger it elicits. Moreover, intermittent fasting regimens, which involve cycles of eating and fasting, might yield different effects on NAD+ levels compared to more extended fasts. Recent research indicates that adopting a dietary approach involving reduced calorie intake and restricting meals to active periods, particularly during daytime hours (i.e., breakfast and lunch), may yield greater benefits for humans compared to consuming food during nighttime hours (7).

## Conclusion

Fasting’s potential to boost NAD+ levels and its implications for combatting viral infections like COVID-19 present an intriguing intersection of ancient practices and modern medical research. The link between NAD+ metabolism, fasting, and host cell responses to viral infections is a complex landscape that necessitates further investigation. Rigorous clinical studies are essential to ascertain the safety, efficacy, and feasibility of fasting regimens as potential adjuvant therapies against viral infections. If harnessed effectively, fasting-induced NAD+ elevation could emerge as a valuable strategy to augment host cell defenses and contribute to managing viral diseases, particularly COVID-19.

## Author contributions

RD: Conceptualization, Writing – review & editing. MM: Conceptualization, Writing – review & editing. CH: Supervision, Writing – review & editing. JS: Conceptualization, Supervision, Writing – original draft, Writing – review & editing.
